# 

**Published:** 1825-01

**Authors:** 


### THE LONDON MEDICAL AND PHYSICAL JOURNAL.

CONTAINING

ORIGINAL CORRESPONDENCE OF EMINENT PRACTITIONERS,

AND

CRITICAL ANALYSIS OF NEW WORKS

RELATING TO MEDICINE, SURGERY, MIDWIFERY, CHEMISTRY, PHARMACY, BOTANY, AND NATURAL HISTORY.

---

EDITED BY

RODERICK MACLEOD, M.D.

LICENTIATE OF THE COLLEGE OF PHYSICIANS OF LONDON; MEMBER OF THE MEDICO-CHIRURGICAL SOCIETY OF LONDON, AND OF THE ROYAL MEDICAL SOCIETY OF EDINBURGH; PHYSICIAN TO THE WESTMINSTER GENERAL DISPENSARY; TO THE INFIRMARY FOR CHILDREN, AND TO THE SCOTTISH HOSPITAL; LECTURER ON THE PRACTICE OF PHYSIC AND MATERIA MEDICA, AT THE MEDICAL THEATRE, WINDMILL-STREET:

AND

JOHN BACOT, Esq.

MEMBER OF THE ROYAL COLLEGE OF SURGEONS; SURGEON TO THE ST. GEORGE'S AND ST. JAMES'S DISPENSARY; AND LATELY SURGEON TO HIS MAJESTY'S GRENADIER REGIMENT OF FOOT GUARDS.

---

VOL. LIII.

FROM JANUARY TO JUNE, 1825.

Et quoniam variant morbi, variabimus artes;
Mille mali species, mille salutis erunt.

---

45576

*LONDON:*

PRINTED FOR THE PROPRIETORS,

*By J. and C. Adlard, Bartholomew Close;*

PUBLISHED BY J. SOUTER, 73, ST. PAUL'S CHURCH-YARD;

AND MAY BE HAD OF ALL BOOKSELLERS.

### MONTHLY CATALOGUE OF MEDICAL BOOKS.

*** *It having been hinted to us that gentlemen, sending copies of their Works, prefer having their titles given at length, it is our intention, in future, to comply with this suggestion: but it is to be observed, that no books can be entered on this List except those sent to us for the purpose;—finding, in the lists hitherto transmitted, that the names of works have frequently been given as published, which have not appeared for weeks, or even months after.*

An Exposition of the Natural System of the Nerves of the Human Body; with the Papers from the Philosophical Transactions on the same Subject. By Charles Bell, Professor of Anatomy and Surgery to the Royal College of Surgeons; Lecturer in the School of Great Windmill-street, and Surgeon to the Middlesex Hospital.—London, 1824.

Report of the Epidemic Cholera, as it has appeared in the Territories subject the Presidency of Fort St. George. Drawn up by order of the Government, under the superintendance of the Medical Board. By William Scot, Surgeon, and Secretary to the Board.—Madras, 1824; folio, pp. lxxii, 292. With an Appendix, containing the Sick Returns of the Army of Fort St. George, from the year 1815 to 1821 inclusive, exhibited in a series of fifteen Tables; also Diurnal Tables of the Progress of Cholera, in six Parts; Tables of the Movements of the Troops, and Meteorological Tables, in seven Parts, from 1815 to 1821.

Medicinisch-Chirurgische Zeitung; September, 1824.

Allgemeine-Medezinische Annalen, &c. &c.; August.

### NOTICE TO CORRESPONDENTS.

*The Communications of* Mr. POLLOCK, Mr. SATH, "A BOROUGH STUDENT," *and* T. K. *have been received.*

Mr. READ's *Communication, having already appeared in the Newspapers, cannot be inserted.*

*As anonymous Correspondents cannot expect the same degree of anthority to be attached to their Communications as to others, we request that Gentlemen will hereafter favour us with their names. We hope this hint will not be lost upon some whose Papers we have just received.*

